# Interventions encouraging the use of systematic reviews by health policymakers and managers: A systematic review

**DOI:** 10.1186/1748-5908-6-43

**Published:** 2011-04-27

**Authors:** Laure Perrier, Kelly Mrklas, John N Lavis, Sharon E Straus

**Affiliations:** 1 Li Ka Shing Knowledge Institute, St. Michael's Hospital; Office of Continuing Education and Professional Development, Faculty of Medicine, University of Toronto, Toronto, Canada; 2 Faculty of Medicine, University of Calgary, Calgary, Canada; 3 McMaster Health Forum, Department of Clinical Epidemiology and Biostatistics, Department of Political Science, McMaster University, Hamilton, Canada; 4 Faculty of Medicine, University of Toronto; Keenan Research Centre, Li Ka Shing Knowledge Institute, St. Michael's Hospital, Toronto, Canada

## Abstract

**Background:**

Systematic reviews have the potential to inform decisions made by health policymakers and managers, yet little is known about the impact of interventions to increase the use of systematic reviews by these groups in decision making.

**Methods:**

We systematically reviewed the evidence on the impact of interventions for seeking, appraising, and applying evidence from systematic reviews in decision making by health policymakers or managers. Medline, EMBASE, CINAHL, Cochrane Central Register of Controlled Trials, Cochrane Methodology Register, Health Technology Assessment Database, and LISA were searched from the earliest date available until April 2010. Two independent reviewers selected studies for inclusion if the intervention intended to increase seeking, appraising, or applying evidence from systematic reviews by a health policymaker or manager. Minimum inclusion criteria were a description of the study population and availability of extractable data.

**Results:**

11,297 titles and abstracts were reviewed, leading to retrieval of 37 full-text articles for assessment; four of these articles met all inclusion criteria. Three articles described one study where five systematic reviews were mailed to public health officials and followed up with surveys at three months and two years. The articles reported from 23% to 63% of respondents declaring they had used systematic reviews in policymaking decisions. One randomised trial indicated that tailored messages combined with access to a registry of systematic reviews had a significant effect on policies made in the area of healthy body weight promotion in health departments.

**Conclusions:**

The limited empirical data renders the strength of evidence weak for the effectiveness and the types of interventions that encourage health policymakers and managers to use systematic reviews in decision making.

## Background

Policymakers and managers working within health systems make decisions in efforts to improve health for individuals. The impact of the choices made by policymakers is experienced in the health status and daily lives of people in the form of laws and regulations, guidelines, public education campaigns, among others [1]. The choices made by healthcare managers affect environments where common goals and strategies must be found between clinical and administrative environments [2]. Overall, decisions by policymakers and managers are made around burdensome health problems, within complex health systems, and ideally involve effective solutions and strategies to support their implementation.

Increasingly, systematic reviews are seen as helpful knowledge support for policymakers and managers [3-6]. Systematic reviews of effects are concise summaries that address sharply defined questions, employing rigorous methods to select credible and relevant information in order to generate summative reports [4,7]. The review was carried out in two stages: 1) a formal scoping review (a method for mapping existing literature in a topic area and identifying gaps [8]) to understand the extent to which evidence from systematic reviews is sought, appraised, understood, and used to inform decision-making in four key areas: clinical practice, health systems management, public health, and policy making; and 2) a systematic review to determine the impact (a change identified by individual perception or by quantification) on professional performance and healthcare outcomes of interventions for seeking, appraising, and applying evidence from systematic reviews in decision making by health policymakers and managers that is reported in this manuscript.

## Methods

### Data sources and searches

The databases of Medline (1950 to April 2010), EMBASE (1980 to April 2010), CINAHL (1982 to April 2010), Cochrane Central Register of Controlled Trials (CENTRAL) (to April 2010), Cochrane Methodology Register (to April 2010), Health Technology Assessment Database (to April 2010), and LISA (Library and Information Science Abstracts) (1969 to April 2010) were searched using the terms systematic review, meta analysis, evidence synthesis, methodologic review, and quantitative review combined with implement, use, utilize, seek, retrieve, appraise, and apply (see Additional File 1). The grey literature was searched after identifying key websites and search engines, such as Google and Intute. Reference lists of all papers and relevant reviews were screened for any further published or unpublished work and experts in the field were contacted to identify any further studies. No language restrictions were placed on the search strategy.

### Study Selection

We included all study designs except qualitative studies. For this study, a health policymaker was defined as an individual elected or appointed to office at some level of government. A health manager was defined as an individual in a managerial or supervisory role, in an institutional healthcare organization with management and supervisory mandates. Both needed to be identified as responsible for decisions on behalf of a large jurisdiction or organization. Studies had to indicate decision makers' use of systematic reviews in either health policy or management decisions, or on a broader range of policy or management decisions if these include health policy or health management decisions in some capacity. Studies of decision making in relation to an individual patient were excluded. Any study that examined interventions intended to increase seeking, appraising, or applying evidence from systematic reviews (as a source document) by a health policymaker or manager was included. The use of products or tools derived from systematic reviews (*e.g.*, guidelines, evidence summaries) was not considered, because our focus was the use of systematic reviews.

Primary outcomes of interest were: the choice to endorse evidence-based problem formulations, programs, and services (and drugs) to address problems; health system arrangements that get effective programs and services to those who need them; and implementation strategies for selected policies, programs and services (*e.g.*, for a tobacco cessation intervention, program, or policy), as well as the choice not to endorse those not supported by the best available evidence by a health policymaker or manager. Two people independently screened all titles and abstracts for inclusion. If at least one person selected the article, it was identified for full-text retrieval.

### Data extraction and quality assessment

Standardized data abstraction forms were developed drawing on the Cochrane EPOC (Effective Practice and Organisation of Care Group) data abstraction form [9] and pilot tested by the review team using the protocol to guide primary and secondary outcomes. The following information was extracted from each article: setting, country, health area addressed, frequency and timing of the intervention, duration of the intervention, format of the intervention (*e.g.*, web-based, person-to-person contact), known effectiveness of the intervention for changing behaviours (*e.g.*, does the study use an evidence-based intervention), nature of the intervention (*e.g.*, training, mode of payment, team approach), number of components included in the intervention, source and authors of the intervention (*e.g.*, professional organization, governmental agency), mode of delivery (*e.g.*, individuals or groups), reliability and validity testing of outcome measurement tools, and adherence (*e.g.*, withdrawals, drop-outs). Two reviewers independently assessed each study and undertook data abstraction directly from primary studies. Disagreements were discussed until consensus was achieved. A third reviewer was available if consensus could not be reached. Authors were contacted for missing data or when clarification was required.

Two independent reviewers assessed the methodological quality of all studies that were included for data abstraction. Any discrepancies in ratings were resolved by discussion. Reviewers were not blinded to study author, institution, or journal, as evidence indicates that little benefit is achieved through blinding [10,11]. The criteria described in section 6.4 of the Data Collection Checklist from the Cochrane EPOC (available at: http://www.epoc.cochrane.org) was used for randomised trials, and a modified Downs and Black tool [12,13] was used for observational studies. The criteria used to assess randomised trials were concealment of allocation, follow up of professionals, follow up of patients or episodes of care, blinded assessment of primary outcome(s), baseline measurement, reliable primary outcome measure(s), and protection against contamination. The criteria used to assess observational studies were reporting, external validity, and internal validity.

## Results

**Figure 1:**
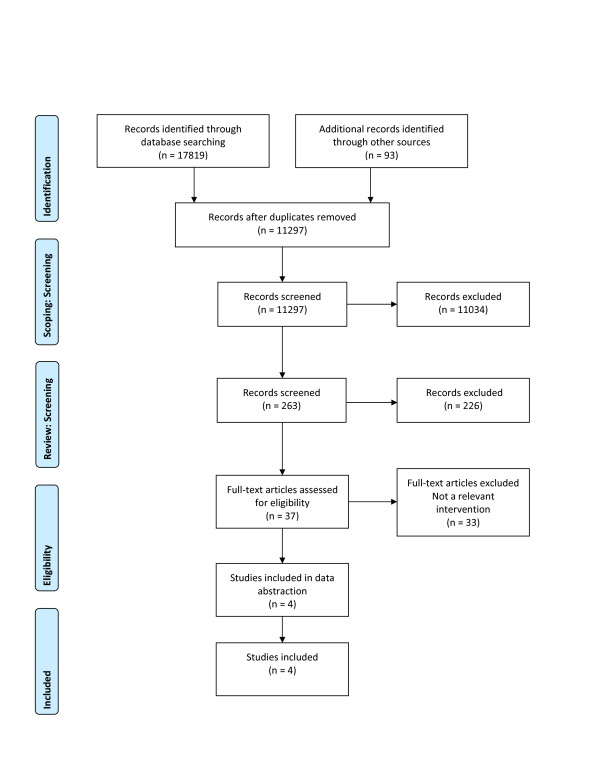
**Flow diagram of systematic review to identify eligible studies**.

Initial searches of electronic databases identified 17,819 records. After removing duplicates, 11,297 records were examined to determine potential relevance. Of these, 263 were identified as related to health policymakers and managers, and 37 full-text articles were retrieved and screened. After screening all studies, 33 articles were excluded due to not having a relevant intervention. Three articles reporting on different aspects of one study that involved two cross-sectional surveys and one article describing a randomised controlled trial met the full inclusion criteria (Figure 1) [14-17].

All studies were identified as focusing on seeking, appraising, and applying evidence from systematic reviews in decision making by policy or managers (Table 1) [14-17]. All surveys took place in Ontario, Canada [14-16].

**Table 1:** Characteristics of included studies

Source	Study Design	Participants and setting	Response rate	Content area of systematic reviews	Intervention	Study outcomes	Quality assessment
Ciliska 1999	Cross-sectional survey	Public health policymakers and managers	Initial survey: 87%	1. Home visiting as a public health intervention	Five systematic reviews disseminated to public health decision makers in 1996	91% requesting systematic review in first survey remembered receiving the information	Inadequate reporting of frequency data
		N = 225	Three-month follow up: 93%	2. Community-based heart health promotion		Of those who remembered, 23% stated it played a part in program planning or decision-making	Discrepancy in number of eligible participants
		Canada		3. Adolescent suicide prevention		57% (of the 23%) reported it influenced actual recommendations made to others - 64% of those recommendations were accepted	Conclusions incongruent with data presented
				4. Community development		Implementation of policies is implied. No specific examples are given	Generalizable only to public health professionals making decisions in Ontario, Canada
				5. Parent-child health			No control group
						Information is self reported	Clustering effect

Dobbins 2001a	Cross-sectional survey	Public health policymakers and managers	Two year follow up: 95.9%	1. Home visiting as a public health intervention	Follow-up to Ciliska 1999 two years later	63% of respondents reported they had used at least one of the systematic reviews in the past 2 years to make a decision	Large number of independent variables with small sample makes interpretation of statistical analysis uncertain
Dobbins 2001b		N = 141		2. Community-based heart health promotion		Implementation of policies is implied. No specific examples are given	Generalizable only to public health professionals making decisions in Ontario, Canada
		Canada		3. Adolescent suicide prevention			No control group
				4. Community development		Information is self reported	Clustering effect
				5. Parent-child health			

Dobbins 2009	Randomised controlled trial	Public health policymakers and managers	108 out of 141 health departments participated in study	Healthy body weight promotion in children	Health department randomised to receive one of three interventions over a period of one year:	No significant effect on global evidence-informed decision-making	The rate of successful intervention may have differed across the three intervention groups due to discrepancies in the ability of interventions to be implemented
		N = 108	Follow up data collected from 88 of 108 health departments		1. access to an online registry of systematic reviews	Significant effect observed for tailored messages plus access to online registry of systematic reviews (*p *< 0.01) in health policies and programs	Investigators were limited by participants' ability to self report
		Canada			2. tailored messages plus access to the online registry of systematic reviews		One representative individual for each organization used to provide data
					3. tailored messages plus access to the registry along with a knowledge broker who worked one-on-one with decision makers		30% of participants had limited engagement with knowledge brokers, thus caution recommended with generalizability.

One randomised trial encouraged health department personnel in Canada to access systematic reviews on healthy body weight promotion in children using one of three potential interventions [17]. A meta-analysis of study outcomes was not possible due to the heterogeneity in the format of the interventions, the settings, and healthcare areas being addressed. It is only feasible to provide a narrative description of the results using a strategy suggested by the Best Evidence Medical Education Collaboration [18] and based on the validity of the individual studies.

### Participants and settings

Public health policymakers and managers were identified as the population examined in all of the studies [14-17]. Ciliska *et al. *[14] described the original research project conducted in 1996, where attempts were made to identify all public health policy decision makers in Ontario, Canada. This was done by contacting the Public Health Branch and every public health department in the province, and asking them to identify all relevant personnel. 270 individuals were identified and invited to take part in the survey. There are discrepancies in reporting, as 277 individuals are later reported as being eligible to participate. Of these, 242 (87%) people completed the first survey and 225 (81%) completed the follow up survey three months later. In 1998, participants were contacted again. They were invited to complete another survey if they had taken part in the previous study and were still employed in a public health department. Of these, 147 participants agreed to participate, and responses were received from 141 participants [15,16]. In the randomised trial, Dobbins *et al. *[17] invited all health departments in Canada to participate with follow-up data obtained from 88 out of 108 departments.

### Interventions

Three articles report on one intervention where public health policymakers are offered the opportunity to receive five relevant systematic reviews in 1996, and followed up at three months [14] and two years [15,16]. The initial survey asked policymakers and managers if they would like to receive a one-time delivery of the five systematic reviews [14]. The systematic reviews offered to the participants covered the public health topics on the effectiveness of: home visiting; community development projects; maternal-child interventions; adolescent suicide prevention; and heart health projects [14-16]. Among other questions, all follow-up surveys specifically asked about the use of the systematic reviews to make a decision related to policy [14-16].

The randomised trial consisted of health departments receiving one of three interventions: access to an online registry of systematic reviews, tailored messages plus access to the online registry of systematic reviews, and tailored messages plus access to the registry along with a knowledge broker who worked one-on-one with decision makers over a period of one year [17]. Data collected for evaluation included effects on global evidence-informed decision making, and effects on public health policies and programs. Global evidence-informed decision making is the extent to which research evidence was considered in recent program-planning decisions related to healthy body weight promotion. Public health policies and programs was a measure derived as a sum of actual strategies, policies, and interventions for healthy body weight promotion in children being implemented by the health department calculated in the timeframe spanning from baseline to post-intervention, which was approximately 18 months.

### Effect of the intervention

Ciliska *et al. *[14] report that three months after the intervention, 91% of participants remembered receiving systematic reviews. Of these, 23% said it played a part in program planning or decision making. Of this group, 57% reported it influenced recommendations made to others, and that 64% of those recommendations were accepted [14]. There is no reporting of examples around how information from the systematic reviews was incorporated into a policy or program [14]. The two articles by Dobbins *et al. *[15,16] describe the survey conducted two years later. Recipients of this survey indicated a 63.1% utilization rate of at least one of the systematic reviews in the two years since they had been in contact. The significant predictors for use of systematic reviews are: the position of the participant -- being a director (OR 9.82, 95% CI 1.48 to 65.32) or manager (OR 14.04, 95% CI 2.22 to 88.96) as compared with medcial and associate medical officers of health; having the expectation to use reviews in future (OR19.25, 95% CI 2.44 to 151.99); having the perception that reviews would overcome limited critical appraisal skills (OR 3.36, 95% CI 1.36 to 8.31); and that reviews were easy to use (OR 3.01, 95% CI 0.98 to 9.29) (Dobbins 2001a). Although 141 people agreed to participate in this survey, only 88 complete surveys were available for statistical analysis that identified these predictors of using systematic reviews. Similar to the reporting by Ciliska *et al. *[14], the implementation of specific policies and programs is reported but no specific examples are given [15,16].

In the randomised trial, Dobbins *et al. *[17] were not able to show a significant effect of any of the interventions on global evidence-informed decision making (*p *< 0.45). With regards to effects on public health policies and programs, health departments that received tailored messages plus access to the online registry of systematic reviews improved significantly from baseline to follow-up (*p *< 0.01) in comparison to the groups that had access to the online registry only, or the groups that had access to the registry and also had a knowledge broker working with them. Research use was further examined by asking participants whether they were in what authors described as 'low' (four of seven on a seven-point Likert scale) versus 'high' (six of seven on a seven-point Likert scale) research cultures within their organizations. They observed that knowledge brokers along with access to systematic reviews showed a trend towards a positive effect when organizational research culture is perceived as low. However, health departments with a low organizational research culture only benefited slightly when they received the tailored message plus access to the online registry of systematic reviews, yet showed great improvements when the research culture was high. These relationships need to be further explored, but they do offer support to the importance of organizational factors.

### Quality Assessment Results

Quality assessments of the studies indicate that clustering effects and other problems that could put them at a risk of bias were identified as sufficient to affect interpretation of results. The paper by Ciliska *et al. *[14] includes limited details on the study design and no details on sample size for the initial follow-up survey. Questions that did not test well during reliability testing were re-worded but not further tested [14]. Dobbins *et al. *[15,16] identify the large number of independent variables combined with a small sample size as a limitation to their second follow-up survey. The authors acknowledge that the large number of independent variables may have resulted in some variables being significant due to chance alone. Thus, the predictors they describe as having a relationship with using systematic reviews, such as the position of the participant (*e.g.*, being a manager or director), must be interpreted with this caution in mind. The lack of independence among subjects within groups (or clustering effects), along with results being generalizable only to public health professionals making decisions in Ontario, Canada were recognized as a limitation of the study. The trial by Dobbins *et al. *[17] describes adequate sequence generation and allocation concealment, and addresses incomplete outcome data. The authors report that there may have been discrepancies in the ability of the interventions to be implemented, and the rate of successful intervention may have differed across the three intervention groups [17]. It is uncertain what effect this has on the study because the interventions were assessed according to group, with the effect being group-specific. Investigators were limited by participants' ability to self report in outcome measures, *e.g.*, research use, as well as the use of one representative individual for each organization to provide data. For the group that worked with a knowledge broker, 30% of participants had limited or no engagement with the knowledge broker, thus the authors recommend caution with the generalizability of these results.

## Discussion

To our knowledge, this is the first systematic review of the literature on the impact of interventions for seeking, appraising, and applying evidence from systematic reviews in decision making by health policymakers or managers. The review of four articles revealed a paucity of experimental research on interventions that encourage policymakers and managers to use systematic reviews in decision making. Three of the articles report data from one intervention that distributed five systematic reviews to health policymakers and managers in public health, with one follow up survey conducted after three months, and another follow-up survey administered two years later. From the two follow-up surveys, authors were able to report that at three months, 23% of participants stated the reviews played a part in program planning or decision making [14]. However, it is not possible to determine if participants did use the results given this is based on self report. Two years later, 63% of respondents reported they had used at least one of the systematic reviews in the past 2 years to make a decision [15,16]. However, data on the proportion of the sample size that responded to these questions as it relates to the original survey by Ciliska *et al. *[14] is not reported, and this lack of context may alter the understanding of results. For instance, one-third of the respondents from the Ciliska *et al. *[14] survey did not participate in this follow-up study two years later [15,16]. Several factors further create challenges in interpreting the data presented in the three articles, including the lack of a control group, methodological limitations relating to small sample size, clustering effect, and limited detail in the reporting of data. The randomised trial suggests that tailored, targeted messages plus online access to systematic reviews can be an effective strategy for evidence-informed decision making.

Several limitations in this review should be considered. The literature in this area is poorly indexed. This challenge was acknowledged in the choice to conduct a scoping review as a strategy to understand the overall state of research activity in the area of the use of systematic reviews in healthcare decision making. Scoping reviews are often undertaken when an area has little published research available, or the area is poorly understood [19]. The search strategy for the scoping review allowed for a very broad search and examination of over 10,000 articles. The small number of studies available for assessment indicates the difficulty in summarizing and identifying key aspects in successful strategies that encourage health policymakers and managers to use systematic reviews in decision making. The limited empirical data render the strength of evidence weak in relation to the effectiveness and the types of interventions that encourage health policymakers and managers to use systematic reviews. Second, this review is limited by the reports of methods from the included studies.

## Conclusions

This review found four relevant articles which provide limited evidence that the interventions outlined changed decision making behaviour. Overall, there is insufficient evidence to support or refute interventions for seeking, appraising, and applying evidence from systematic reviews in decision making by health policymakers and managers, however the intervention describing the use of tailored messages is promising. Considerations for future research include examining the circumstances and contexts under which systematic reviews are most effective. This includes how systematic reviews are accessed, when they are used (*e.g.*, different points in the process of developing policies), identifying the types of reviews needed in concert with the stage of policymaking (effectiveness versus process evaluation), understanding more about the local applicability of systematic reviews, and the specific characteristics that make systematic reviews easy to use in terms of presentation and format of information (*e.g.*, grading entries, providing contextual information) [5,20-22].

## Competing interests

The authors declare that they have no competing interests.

## Authors' contributions

SES created the study concept and design. LP and SES constructed and refined the search strategy. SES and LP acquired the data. Analysis and interpretation of the data was completed by LP, KM, and SES. Drafting of the manuscript and critical revision for important intellectual content was done by LP, KM, SES, and JNL. LP wrote the final report and is the guarantor for the paper. All authors read and approved the final manuscript.

## Supplementary Material

Additional file 1 Medline search strategy to identify studies. Search strategy performed in OVID Medline^®^. Click here for file
