# Supplementary material for: Interventions encouraging the use of systematic reviews by health policymakers and managers: A systematic review
Source: Implement Sci. 2011 Apr 27;6:43. doi: 10.1186/1748-5908-6-43 (PMC3104485; doi:10.1186/1748-5908-6-43)
Supplement: Additional file 1 — Medline search strategy to identify studies. Search strategy performed in OVID Medline®. [file 1748-5908-6-43-S1.PDF]

Database: Ovid MEDLINE(R) <1950 to April Week 2 2010>

Search Strategy:

1 (systematic review? adj3 (implement$ or using or use? or seek$ or sought or retriev$ or apprais$ or appl$ or utiliz$ or utilis$ or operational$ or interpret$ or execut$ or administ$ or practi?e?)).tw.

2 (systematic overview? adj3 (implement$ or using or use? or seek$ or sought or retriev$ or apprais$ or appl$ or utiliz$ or utilis$ or operational$ or interpret$ or execut$ or administ$ or practi?e?)).tw.

3 (meta-analys$ adj3 (implement$ or using or use? or seek$ or sought or retriev$ or apprais$ or appl$ or utiliz$ or utilis$ or operational$ or interpret$ or execut$ or administ$ or practi?e?)).tw.

4 (metaanalys$ adj3 (implement$ or using or use? or seek$ or sought or retriev$ or apprais$ or appl$ or utiliz$ or utilis$ or operational$ or interpret$ or execut$ or administ$ or practi?e?)).tw.

5 (metanalys$ adj3 (implement$ or using or use? or seek$ or sought or retriev$ or apprais$ or appl$ or utiliz$ or utilis$ or operational$ or interpret$ or execut$ or administ$ or practi?e?)).tw.

6 (meta-regression adj3 (implement$ or using or use? or seek$ or sought or retriev$ or apprais$ or appl$ or utiliz$ or utilis$ or operational$ or interpret$ or execut$ or administ$ or practi?e?)).tw.

7 (metaregression adj3 (implement$ or using or use? or seek$ or sought or retriev$ or apprais$ or appl$ or utiliz$ or utilis$ or operational$ or interpret$ or execut$ or administ$ or practi?e?)).tw.

8 (meta-ethnograph$ adj3 (implement$ or using or use? or seek$ or sought or retriev$ or apprais$ or appl$ or utiliz$ or utilis$ or operational$ or interpret$ or execut$ or administ$ or practi?e?)).tw.

9 (metaethnograph$ adj3 (implement$ or using or use? or seek$ or sought or retriev$ or apprais$ or appl$ or utiliz$ or utilis$ or operational$ or interpret$ or execut$ or administ$ or practi?e?)).tw.

10 (meta-synthes$ adj3 (implement$ or using or use? or seek$ or sought or retriev$ or apprais$ or appl$ or utiliz$ or utilis$ or operational$ or interpret$ or execut$ or administ$ or practi?e?)).tw.

11 (metasynthes$ adj3 (implement$ or using or use? or seek$ or sought or retriev$ or apprais$ or appl$ or utiliz$ or utilis$ or operational$ or interpret$ or execut$ or administ$ or practi?e?)).tw.

12 (cochrane adj3 (implement$ or using or use? or seek$ or sought or retriev$ or apprais$ or appl$ or utiliz$ or utilis$ or operational$ or interpret$ or execut$ or administ$ or practi?e?)).tw.

13 (evidence synthesis adj3 (implement$ or using or use? or seek$ or sought or retriev$ or apprais$ or appl$ or utiliz$ or utilis$ or operational$ or interpret$ or execut$ or administ$ or practi?e?)).tw.

14 (quantitative review adj3 (implement$ or using or use? or seek$ or sought or retriev$ or apprais$ or appl$ or utiliz$ or utilis$ or operational$ or interpret$ or execut$ or administ$ or practi?e?)).tw.

15 (quantitative overview adj3 (implement$ or using or use? or seek$ or sought or retriev$ or apprais$ or appl$ or utiliz$ or utilis$ or operational$ or interpret$ or execut$ or administ$ or practi?e?)).tw.

16 (methodologic$ review adj3 (implement$ or using or use? or seek$ or sought or retriev$ or apprais$ or appl$ or utiliz$ or utilis$ or operational$ or interpret$ or execut$ or administ$ or practi?e?)).tw.

17 (methodologic$ overview adj3 (implement$ or using or use? or seek$ or sought or retriev$ or apprais$ or appl$ or utiliz$ or utilis$ or operational$ or interpret$ or execut$ or administ$ or practi?e?)).tw.

18 (qualitative review adj3 (implement$ or using or use? or seek$ or sought or retriev$ or apprais$ or appl$ or utiliz$ or utilis$ or operational$ or interpret$ or execut$ or administ$ or practi?e?)).tw.

19 (qualitative overview adj3 (implement$ or using or use? or seek$ or sought or retriev$ or apprais$ or appl$ or utiliz$ or utilis$ or operational$ or interpret$ or execut$ or administ$ or practi?e?)).tw.

20 (technology assessment? adj3 (implement$ or using or use? or seek$ or sought or retriev$ or apprais$ or appl$ or utiliz$ or utilis$ or operational$ or interpret$ or execut$ or administ$ or practi?e?)).tw

21 *"Review Literature as Topic"/ and (implement$ or seek$ or sought or retriev$ or apprais$ or appl$ or utiliz$ or utilis$ or operational$ or interpret$ or execut$ or administ$).tw.

22 *Meta-Analysis as Topic/ and (implement$ or seek$ or sought or retriev$ or apprais$ or appl$ or utilis$ or utiliz$ or operational$ or interpret$ or execut$ or administ$).tw.

23 or/1-22
